# The effects of climate factors on scabies. A 14-year population-based study in Taiwan

**DOI:** 10.1051/parasite/2016065

**Published:** 2016-12-01

**Authors:** Jui-Ming Liu, Hsiao-Wei Wang, Fung-Wei Chang, Yueh-Ping Liu, Feng-Hsiang Chiu, Yi-Chun Lin, Kuan-Chen Cheng, Ren-Jun Hsu

**Affiliations:** 1 Division of Urology, Department of Surgery, Taoyuan General Hospital, Ministry of Health and Welfare 330 Taoyuan Taiwan; 2 Division of Infectious Diseases, Department of Internal Medicine, School of Medicine, College of Medicine, Taipei Medical University 110 Tapei Taiwan; 3 Division of Infection Diseases, Department of Internal Medicine, Shuang Ho Hospital, Taipei Medical University 235 Tapei Taiwan; 4 Department of Obstetrics & Gynecology, Tri-Service General Hospital, National Defense Medical Center 114 Tapei Taiwan; 5 Department of Emergency Medicine, National Taiwan University Hospital 100 Tapei Taiwan; 6 Department of Emergency Medicine, Shuang Ho Hospital, Taipei Medical University 235 Tapei Taiwan; 7 Superintendent Office, Ningbo Medical Center, Lihuili Eastern Hospital Ningbo City 315000 Zhejiang Province PR China; 8 Department of Internal Medicine, Lo-Hsu Medical Foundation, Lotung Poh-Ai Hospital 265 Luodong Taiwan; 9 Graduate Institute of Food Science and Technology, National Taiwan University 106 Tapei Taiwan; 10 Institute of Biotechnology, National Taiwan University 106 Tapei Taiwan; 11 Graduate Institute of Life Sciences, National Defense Medical Center 114 Tapei Taiwan; 12 Department of Pathology and Graduate Institute of Pathology and Parasitology, the Tri-Service General Hospital, National Defense Medical Center 114 Tapei Taiwan; 13 Biobank Management Center of the Tri-Service General Hospital, National Defense Medical Center 114 Tapei Taiwan

**Keywords:** Climate, Scabies, Taiwan, Population studies, National Health Insurance Research Database

## Abstract

Scabies is a common infectious disease and can cause severe outbreaks if not controlled quickly. Besides personal contact history, environmental factors are also important. This study analyzed the effects of environmental climate factors on the incidence of scabies in Taiwan. We conducted a 14-year nationwide population-based study: a total of 14,883 patients with scabies infestation were enrolled. Monthly climate data were collected from Taiwan’s Central Weather Bureau, including data on temperature, relative humidity, total rainfall, total rain days, and total sunshine hours. The linear relationships between these climate factors and scabies infestations or other risk factors were examined by Pearson’s correlation analysis. Overall, the incidence of scabies was negatively correlated with temperature (*γ* = −0.152, *p* < 0.001), while being positively correlated with humidity (*γ* = 0.192, *p* < 0.001). This useful information may provide evidence for lowering humidity at nursing facilities, hospitals, and military camps with scabies infestations, which may help to reduce its spread and prevent outbreaks.

## Abstract (fr)

La gale est une maladie infectieuse courante et peut causer des épidémies sévères si elle n’est pas contrôlée rapidement. Outre l’historique de contact personnel, les facteurs environnementaux sont également importants. Cette étude a analysé les effets des facteurs climatiques environnementaux sur l’incidence de la gale à Taiwan. Nous avons mené une étude nationale de 14 ans sur la population : un total de 14883 patients atteints d’infestation par la gale ont été enrôlés. Les données mensuelles sur le climat ont été recueillies auprès du Bureau Central Météorologique de Taiwan, y compris la température, l’humidité relative, les précipitations totales, le total des jours de pluie et le total d’heures d’ensoleillement. Les relations linéaires entre ces facteurs climatiques et les infestations de gale ou d’autres facteurs de risque ont été examinées par l’analyse de corrélation de Pearson. Dans l’ensemble, l’incidence de la gale était corrélée négativement à la température (*γ* = −0.152, *p* < 0.001), tout en étant positivement corrélée à l’humidité relative (*γ* = 0.192, *p* < 0.001). Cette information utile peut fournir une indication pour abaisser l’humidité dans les établissements de soins infirmiers, les hôpitaux et les camps militaires avec des infestations de gale, ce qui peut aider à réduire sa propagation et prévenir les épidémies.

## Introduction

Scabies is an infectious disease that is common around the world, with a global prevalence of about 300 million cases (or about 5% of the world’s population) per year [15]. Scabies is a kind of parasitic infection caused by the mite *Sarcoptes scabiei* variety *hominis*. Scabies is mostly transmitted by person-to-person contact, and many outbreaks have been reported in nursing homes, hospitals, and army facilities [5, 20]. All persons who have had contact history with infected patients, especially family members or those who have engaged in sexual contact, should receive preventative treatment [10] since asymptomatic infestation is likely to lead to re-infestation of the index case who is highly symptomatic. As such, scabies infestations increase financial burden in terms of medical resources and public health efforts. Various types of people have been reported to be more susceptible to scabies infestations, including people who live in urban areas, immunocompromised persons, the elderly, and institutionalized patients [12, 29]. Complications of scabies may also be found due to secondary bacterial infections [22].

Meanwhile, a variety of environmental factors also influence human health. Seasonal variations have been observed for a number of clinical diseases, including myocardial infarction and several infectious diseases [14]. Seasonal variations in the incidences of infectious diseases may be associated with the epidemiology of the prevalent pathogens, changes in environmental and meteorological parameters, and alterations in human behavior. Scabies infestations have also been found to exhibit seasonal variations, with infections being especially common in the winter [12].

Although previous studies have revealed seasonal fluctuations in the rate of scabies infestations [20], there is a lack of studies regarding how scabies infestations are affected by different climates. In the present study, we aimed to investigate the effect of various climate variables, such as temperature, humidity, and total rainfall, on the incidence of scabies infestations. To do so, we conducted a 14-year retrospective population-based study to evaluate the relationship between climate parameters and scabies.

## Materials and methods

### Data sources and collection

We conducted a nationwide population-based study using data from Taiwan’s National Health Insurance Research Database (NHIRD). The NHIRD collects data from the National Health Insurance (NHI) program, which started in 1995 and covered 99.9% of the 23 million people in Taiwan as of the end of 2013 [18]. The NHIRD contains all the medical records of inpatients and outpatients, including demographic data such as sex, date of birth, location, and insured amount, as well as medical records of clinical visits, admissions, and clinical procedures. Specifically, we used the Longitudinal Health Insurance Database 2000 (LHID2000), a sub-dataset of the NHIRD, for this study. The LHID2000 contains data from January 2000 to December 2013 for a randomly selected sample of one million people out of the 23 million people included in the NHIRD in 2000 [9]. The LHID2000 and NHIRD have a similar demographic distribution and origin of population [21]. The clinical diagnoses were made by the International Classification of Diseases, 9th revision, Clinical Modification (ICD-9-CM) [27]. The weather data in this study were collected by Taiwan’s Central Weather Bureau (CWB). The CWB monitors 27 weather stations throughout Taiwan, which includes the islands of Taiwan, Penghu, Kinmen, and Lienchiang. We used monthly weather data in this study, including data on temperatures (measured in degrees Celsius (°C)), relative humidity (recorded as a percentage (%)), total rainfall (measured in millimeters (mm)), total rain days (recorded in days), and total sunshine hours (recorded in hours). In Taiwan, the spring season includes March, April, and May; the summer season includes June, July, and August; the fall season includes September, October, and November; and the winter season includes December, January, and February.

### Ethics

This study was approved by the Institutional Review Board of the Tri-Service General Hospital (approval number: TSGHIRB NO. B-105-06). As this was a retrospective study and all data were anonymous, the Institutional Review Board department agreed that it was not necessary to obtain patient consent.

### Study population

**Figure 1.**
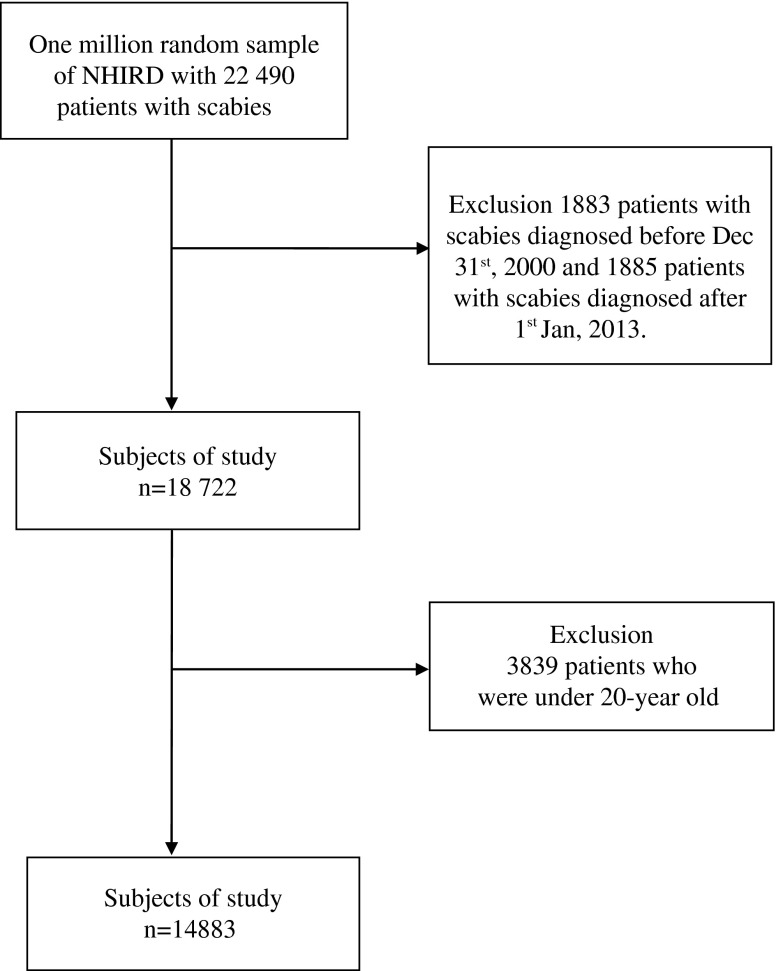
Flowchart of collection of study subjects of National Health Insurance Research Database (NHIRD), from January 2000 to December 2013.

The study subjects were selected from the LHID2000 covering the period from January 2000 to December 2013. The selected study subjects were newly diagnosed with scabies infestation (ICD-9-CM 133.0) between 2000 and 2013 (Fig. 1). All such diagnoses of scabies infestations were made by physicians, and all the patients were prescribed anti-scabies oral or topical medication for treatment.

The exclusion criteria for the study were as follows: patients who were diagnosed before December 31, 2000, or after January 1, 2013 (*n* = 3768); and patients who were younger than 20 years old (*n* = 3839). In Taiwanese civil law, people in their 20th year are considered adults. In general, study populations of adult are able to be approved by the Institutional Review Board in Taiwan. Studies in populations under 20 years of age are difficult to approve by Institutional Review Boards in Taiwan. Given these criteria, there were a total of 14,883 patients with scabies infestations who were enrolled in this study. A given diagnosis of scabies was made according to the patient’s history and a physical examination by a licensed physician. Typical physical examination findings for those diagnosed with scabies include the following: generalized itching sparing the face and head; severe pruritus at night; inflammatory pruritic papules at infected body sites, especially the finger webs, flexor part of the wrists, flexor part of the elbows, axillae, buttocks, genitalia, and (in female patients) the breasts; and burrows and nodules [10].

### Variables

The variables in this study were chosen on the basis of related studies in the past literature. Specifically, we selected the following variables: chronic pulmonary disease (ICD-9-CM 490-492, 494,496), diabetes mellitus (ICD-9-CM 250), hypertension (ICD-9-CM 401.1, 401.9), peptic ulcer disease (ICD-9-CM 533), mild liver disease (ICD-9-CM 571.2, 571.4–571.6), congestive heart failure (ICD-9-CM 428), rheumatologic disease (ICD-9-CM 714, 725-729), cerebrovascular disease (ICD-9-CM 430-438), malignancy (ICD-9-CM 140-208), Parkinson disease (ICD-9-CM 332), tuberculosis (ICD-9-CM 011,012), chronic kidney disease (ICD-9-CM 585,586,588), liver cirrhosis (ICD-9-CM: 571), hyperlipidemia (ICD-9-CM 272.4), heart disease (ICD-9-CM 393–398, 402, 404.0, 404.1, 404.9, 410–414, 415.0, 416.1, 416.8, 416.9, 420–429), anemia (ICD-9-CM 280–285), and epilepsy (ICD-9-CM 345). The monthly income levels of the study subjects were also taken from NHI program data and were categorized into the following four groups: <20,000 new Taiwan dollars (NTD); 20,000–40,000 NTD; 40,000–60,000 NTD; and ≥60,000 NTD.

### Statistical analysis

Descriptive statistical data for the study subjects and weather factors were calculated by the Microsoft^®^ SQL Server^®^ 2008. Further data analysis was conducted using IBM SPSS statistics software, version 20 (IBM SPSS, 2013). Linear correlations between the weather factors and the incidence of scabies were calculated by Pearson’s correlation method. A two-sided *p*-value < 0.05 was regarded as statistically significant in all statistical tests.

### Results

A total of 14,883 patients diagnosed with scabies infestations from 2000 to 2013 in Taiwan were included in this study. The demographic characteristics and medical conditions of the study subjects are shown in Table 1. The mean age of the patients with scabies was 52.4 ± 21.0 years, and two age groups were predominant: patients older than 70 years old (26.9%) and patients 20–29 years old (19.0%). There were slightly more male patients than female patients, and the mean CCI index was 1.4 ± 2.0. The comorbidities most frequently linked with scabies infestations were hypertension, heart disease, chronic pulmonary disease, cerebrovascular disease, peptic ulcer disease, and diabetes. Patients with an insured amount between 20,000 and 40,000 NTD represented a higher percentage of the total (51.4%) than those in other insured amount ranges.

**Table 1.** Demographic characteristics of patients with scabies in Taiwan, from 2000 to 2013.

Characteristics	Scabies infected cases, *n*	%
No. of cases	14 883 (100.0%)	
Age (mean ± SD, years)	52.4 ± 21.0	
20–29	2831	19.0
30–39	2190	14.7
40–49	2259	15.2
50–59	2065	13.9
60–69	1537	10.3
≥70	4001	26.9
Sex		
Female	7026	47.2
Male	7857	52.8
Comorbidity disease*		
Chronic pulmonary disease	3054	20.5
Diabetes	2396	16.1
Hypertension	4905	33.0
Peptic ulcer disease	2502	16.8
Mild liver disease	1113	7.5
Congestive heart failure	808	5.4
Rheumatologic disease	667	4.5
Cerebrovascular disease	2773	18.6
Malignancy	572	3.8
Parkinson disease	808	5.4
Tuberculosis	522	3.5
Chronic kidney disease	223	1.5
Liver cirrhosis	1294	8.7
Hyperlipidemia	901	6.1
Heart disease	3672	24.7
Anemia	1169	7.9
Epilepsy	420	2.8
Insured amount (NTD)		
<20,000	4385	29.5
20,000–40,000	7656	51.4
40,000–60,000	2012	13.5
≧60,000	830	5.6
Insured region in Taiwan		
Northern	6569	44.1
Middle	3374	22.7
Southern	4363	29.3
Eastern and outlying islands	577	3.9

* Any kind of 17 comorbidities.

NTD = New Taiwan Dollar USD/TWD≒33.

The average monthly mean data for the various climate factors from 2000 to 2013 are shown in Table 2. Taiwan is located in the Western Pacific and has a subtropical climate. The climate data analyzed for this study included data on temperatures (°C), total sunshine hours, relative humidity (%), total rainfall (mm), and total rain days. The average monthly temperature was lowest (16.9 °C) in January and highest (28.72 °C) in July. The average total sunshine hours were lowest (125.81) in February and highest (223.84) in July. The average monthly relative humidity in Taiwan ranged between 73% and 79%. The average total rainfall and total rain days were both highest in the months of June, July, and August.

**Table 2.** Average monthly mean meteorological factors in Taiwan from 2000 to 2013.

Month	Temperature (ºC)			Total sunshine hours	Relative humidity (%)	Total rainfall (mm)	Total rain days
	Mean	Max	Minimum				
January	16.90	21.90	10.60	128.48	75.95	62.23	8.29
February	18.17	23.50	10.80	125.81	77.64	66.78	7.78
March	19.79	24.30	12.80	139.23	75.42	77.97	9.50
April	22.94	26.80	17.10	128.83	77.24	108.26	10.84
May	25.86	28.70	20.50	160.26	77.85	213.51	12.09
June	27.52	29.80	21.60	167.20	79.31	315.79	13.80
July	28.72	30.80	22.50	223.84	77.14	291.61	11.28
August	28.48	30.10	22.30	196.28	78.31	347.25	13.52
September	27.39	30.00	21.20	171.50	77.44	283.08	11.52
October	24.92	28.00	19.00	175.30	73.97	101.41	6.51
November	22.06	25.60	16.90	139.00	75.03	95.65	7.87
December	18.41	22.90	11.60	137.63	73.73	71.05	7.39

Correlations between the seasonal climate parameters and scabies from 2000 to 2013 in Taiwan are shown in Table 3. Overall, two climate parameters were significantly associated with the incidence of scabies. Specifically, average temperature was negatively correlated with the incidence of scabies (*γ* = −0.152, *p* < 0.001), while relative humidity was positively correlated with the incidence of scabies (*γ* = 0.192, *p* < 0.001). Table 3 also shows the relationships between the climate parameters and the incidence rates of scabies for the different seasons of the year. In spring, average temperature was negatively correlated with the incidence of scabies (*γ* = −0.262, *p* < 0.001), while total rainfall was positively correlated with the incidence of scabies (*γ* = 0.212, *p* = 0.011). In summer, the incidence of scabies was negatively correlated with total sunshine hours (*γ* = −0.305, *p* < 0.001) and positively correlated with total rain days (*γ* = 0.190, *p* = 0.022). In fall, the incidence of scabies was positively correlated with relative humidity (*γ* = 0.217, *p* = 0.009). In winter, the incidence of scabies was negatively correlated with temperature (*γ* = −0.336, *p* < 0.001) and positively correlated with relative humidity (*γ* = 0.475, *p* < 0.001) and total rainfall (*γ* = 0.317, *p* < 0.001).

**Table 3.** Correlation of seasonal climate parameters to scabies from 2000 to 2013 in Taiwan.

Average monthly climate parameters	Incidence of scabies infection	
	*r*	*p*
All		
Temperature (°C)	−0.152	<0.001***
Total sunshine hours	−0.059	0.155
Relative humidity (%)	0.192	<0.001***
Total rainfall amount (mm)	0.034	0.416
Total rain days	0.046	0.272
Spring		
Temperature (°C)	−0.262	0.001**
Total sunshine hours	0.041	0.629
Relative humidity (%)	0.119	0.154
Total rainfall amount (mm)	0.212	0.011*
Total rain days	0.094	0.261
Summer		
Temperature (°C)	0.126	0.133
Total sunshine hours	−0.305	<0.001***
Relative humidity (%)	−0.045	0.593
Total rainfall amount (mm)	0.098	0.241
Total rain days	0.190	0.022*
Fall		
Temperature (°C)	−0.143	0.087
Total sunshine hours	0.059	0.484
Relative humidity (%)	0.217	0.009*
Total rainfall amount (mm)	−0.055	0.512
Total rain days	−0.022	0.792
Winter		
Temperature (°C)	−0.336	<0.001***
Total sunshine hours	0.035	0.674
Relative humidity (%)	0.475	<0.001***
Total rainfall amount (mm)	0.317	<0.001***
Total rain days	0.082	0.328

*r*: Pearson’s correlation, ***p < 0.001; **p = 0.001; *p < 0.05.

## Discussion

This is the first study to investigate the relationships between different climate parameters and the incidence of scabies in Taiwan. The results of this 14-year nationwide population-based study showed that the incidence of scabies was negatively correlated with average temperature and positively correlated with relative humidity.

Taiwan lies in the Western Pacific at the Tropic of Cancer, and the general climate of Taiwan ranges from marine subtropical to tropical. Temperature has been well established by previous research studies to be among the climate factors that influence the incidence of scabies. Scabies is a kind of parasitic infection caused by the mite *Sarcoptes scabiei*. Higher temperatures accelerate the desiccation and death of these mites [13]. In contrast, the mites have better survival and higher fertility rates in cool weather [4, 24], with the mite eggs being capable of remaining viable off of a host for up to 10 days at low temperatures [3]. Long-term observations from Scotland and Israel have demonstrated that the incidence of scabies in those countries was higher during cooler seasons, indicating that the increased person-to-person contact and overcrowding that occur in colder weather facilitate the spread of scabies [3, 20]. Consistent with these studies, our study also demonstrated a negative correlation between temperature and the rate of scabies infestations.

Previous studies have found that scabies mites can survive much longer (up to 19 days) in a cool and humid environment than under general indoor conditions (only 1.5 days) [3]. Relatedly, our study found that relative humidity was positively correlated with the rate of scabies infestations in Taiwan, implying that humid environments result in higher rates of scabies infestations.

Although we did not observe a significant seasonality trend in the incidence of scabies, seasonality trends for scabies infestations have been reported in a large population study of the Israeli Army (specifically, this study reported a higher incidence in winter than in summer) [20]. Many studies, in fact, have reported high incidences of scabies infestations during the cool weather of the fall and winter seasons [1, 25, 26]. As such, we further investigated the relationships between climate parameters and scabies infestations according to the different seasons. In spring, average temperature was negatively correlated with the incidence of scabies, while total rainfall was positively correlated with the incidence of scabies. In fall, relative humidity was positively correlated with the incidence of scabies infestations. In winter, temperature was negatively correlated with the incidence of scabies, whereas relative humidity and total rainfall were positively correlated with the incidence of scabies. Taken together, these results suggest that the cool and humid weather typical of the aforementioned seasons was more suitable for the survival and fertility of the scabies mites. In summer, meanwhile, the incidence of scabies was positively correlated with total rainfall days and negatively correlated with total sunshine hours. Perhaps relatedly, human behaviors also play an important role in the incidence of scabies infestations. For example, the higher number of rainy days in summer in Taiwan may cause people to stay inside, causing overcrowding and higher levels of contact with other people. This increased personal contact may, in turn, tend to result in more scabies infestations.

In our study, two age groups were predominant: patients older than 70 years of age and patients aged 20–29 years. A previous study in Northern Taiwan found that elderly patients with certain risk factors, such as being bedridden, living in a nursing home, poor clinical status on admission, and long-term use of a catheter, had higher rates of scabies infestations [28]. Other studies have also reported that immunocompromised persons, the elderly, and institutionalized patients face greater risks of scabies infestation [12, 29]. In underdeveloped countries, the incidence of scabies is higher in younger populations, whereas the prevalence is similar across all ages in developed nations [6, 8]. In Taiwan, most young male adults perform compulsory military service in their early 20s, meaning that they typically live together with other young males for one to two years. Perhaps related to this, the term “army itch” has been used in various countries for more than 100 years, with cases of this disease erupting during times of war. Investigations by experts have found that scabies infestations are the cause of this so-called “army itch” [11]. Related studies have also found that scabies infestations are particularly prevalent among those engaged in military service, especially in times of war [19, 23]. Moreover, the normal social activities of young infected adults, such as visits home or sexual activities, may facilitate the spread of scabies and cause community outbreaks [16, 19].

Scabies may be difficult to diagnose at the beginning of infection. Scabies infestation may be misdiagnosed as an adverse drug reaction [2], atopic dermatitis [17], contact dermatitis [17], Langerhans cell histiocytosis [7], or immunobullous disease [17]. Moreover, misdiagnoses may lead to serious consequences or even outbreaks. In this study, misdiagnoses of scabies may exist, leading to underestimation of the incidence of scabies. Even skin biopsy may also involve the possibility of misdiagnosis [2]. The diagnosis should be made on the basis of patient history and detailed physical examination of skin lesions. Definitive diagnosis requires the microscopic identification of mites or mite parts.

There are some limitations to this study. First, this is a retrospective population-based study. Second, the diagnoses of scabies among the study subjects were based only on physical examinations and patient histories, and young physicians may have made some misdiagnoses due to lack of experience. Third, the data for the climate parameters investigated were from 27 weather stations monitored by Taiwan’s CWB, but these 27 stations did not cover every village and town in Taiwan. Given these limitations, further large prospective randomized studies are needed to investigate the relationships between climate parameters and scabies infestations.

Scabies is a worldwide problem. While scabies is a treatable disease with a low fatality rate, it is also highly contagious. Fortunately, early recognition of scabies can reduce the length of hospitalization, reduce ward closures for infection control, allow for early treatment of patients, and limit the cost of environmental disinfection procedures. In conclusion, this study found that average temperature was negatively correlated with the incidence of scabies, while relative humidity was positively correlated with incidence. As such, lowering humidity and elevating room temperature may help to reduce rates of scabies infestation. Accordingly, we suggest that room temperatures be raised while humidity levels are lowered in the event that suspected scabies patients are found in nursing facilities, hospitals, and military camps in order to potentially help prevent further spread of the disease. Knowledge of the climate factors predicting increased incidences of scabies may help health policy planners and physicians to release alerts under certain climate conditions, decrease the disease burden, and prevent further outbreaks in high-risk environments.

## Conflict of interest

The authors declare no conflict of interest in relation with this paper.

## References

[1] Ahmed S, Aftabuddin AK. 1977 Common skin diseases (analysis of 7636 cases). Bangladesh Medical Research Council Bulletin, 3(1), 41–45. 615580

[2] Almond DS, Green CJ, Geurin DM, Evans S. 2000 Lesson of the week: Norwegian scabies misdiagnosed as an adverse drug reaction. British Medical Journal, 320(7226), 35–36. 10617527 10.1136/bmj.320.7226.35 PMC1117313

[3] Arlian LG, Runyan RA, Estes SA. 1984 Cross infestivity of *Sarcoptes scabiei*. Journal of the American Academy of Dermatology, 10(6), 979–986. 6736342 10.1016/s0190-9622(84)80318-7

[4] Arlian LG, Runyan RA, Achar S, Estes SA. 1984 Survival and infectivity of *Sarcoptes scabiei* var *canis* and var *hominis*. Journal of the American Academy of Dermatology, 11(2 Pt 1), 210–215. 6434601 10.1016/s0190-9622(84)70151-4

[5] Barrett NJ, Morse DL. 1993 The resurgence of scabies. Communicable disease report. CDR review, 3(2), 32–34. 7693143

[6] Blumenthal DS, Taplin D, Schultz MG. 1976 A community out-break of scabies. American Journal of Epidemiology, 104(6), 667–672. 998612 10.1093/oxfordjournals.aje.a112345

[7] Burch JM, Krol A, Weston WL. 2004 *Sarcoptes scabiei* infestation misdiagnosed and treated as Langerhans cell histiocytosis. Pediatric Dermatology, 21(1), 58–62. 14871329 10.1111/j.0736-8046.2004.21113.x

[8] Burkart CG. 1983 Scabies: an epidemiologic reassessment. Annals of Internal Medicine, 98(4), 498–503. 6340578 10.7326/0003-4819-98-4-498

[9] Chang FW, Lee WY, Liu YP, Yang JJ, Chen SP, Cheng KC, Lin YC, Ho TW, Chiu FH, Hsu RJ, Liu JM. 2016 The relationship between economic conditions and postpartum depression in Taiwan: a nationwide population-based study. Journal of Affective Disorders, 204(1), 174–179. 27362733 10.1016/j.jad.2016.06.043

[10] Chosidow O. 2006 Scabies. New England Journal of Medicine, 354(16), 1718–1727. 16625010 10.1056/NEJMcp052784

[11] Cropley TG. 2006 The “army itch:” A dermatological mystery of the American civil War. Journal of the American Academy of Dermatology, 55(2), 302–303. 16844515 10.1016/j.jaad.2006.04.030

[12] Downs AM, Harvey I, Kennedy CT. 1999 The epidemiology of head lice and scabies in the UK. Epidemiology & Infection, 122(3), 471–477. 10459652 10.1017/s0950268899002277 PMC2809643

[13] Estes SA, Estes J. 1993 Therapy of scabies: nursing homes, hospitals, and the homeless. Seminars in Dermatology, 12(1), 26–33. 7682834

[14] Falagas ME, Theocharis G, Spanos A, Vlara LA, Issaris EA, Panos G, Peppas G. 2008 Effect of meteorological variables on the incidence of respiratory tract infections. Respiratory Medicine, 102(5), 733–737. 18242070 10.1016/j.rmed.2007.12.010

[15] Hengge UR, Currie BJ, Jäger G, Lupi O, Schwartz RA. 2006 Scabies: a ubiquitous neglected skin disease. Lancet Infectious Diseases, 6(12), 769–779. 17123897 10.1016/S1473-3099(06)70654-5

[16] Heukelbach J, Wilcke T, Winter B, Feldmeier H. 2005 Epidemiology and morbidity of scabies and pediculosis capitis in resource-poor communities in Brazil. British Journal of Dermatology, 153(1), 150–156. 16029341 10.1111/j.1365-2133.2005.06591.x

[17] Hicks MI, Elston DM. 2009 Scabies. Dermatology and Therapy, 22(4), 279–292. 10.1111/j.1529-8019.2009.01243.x 19580575

[18] Liu JM, Lin PH, Hsu RJ, Chang YH, Cheng KC, Pang ST, Lin SK. 2016 Complementary traditional Chinese medicine therapy improves survival in patients with metastatic prostate cancer. Medicine (Baltimore), 95(31), e4475. 27495088 10.1097/MD.0000000000004475 PMC4979842

[19] Mimouni D, Gdalevich M, Mimouni FM, Haviv J, Ashkenazi I. 1998 The epidemiologic trends of scabies among Israeli soldiers: a 28-year follow-up. International Journal of Dermatology, 37(8), 586–587. 9732002 10.1046/j.1365-4362.1998.00491.x

[20] Mimouni D, Ankol OE, Davidovitch N, Gdalevich M, Zangvil E, Grotto I. 2003 Seasonality trends of scabies in a young adult population: a 20-year follow-up. British Journal of Dermatology, 149(1), 157–159. 12890210 10.1046/j.1365-2133.2003.05329.x

[21] National Health Research Institutes. 2015 National Health Insurance Research Database (online). Available at: http://nhird.nhri.org.tw/en/Data_Subsets.html#S3 Accessed October 1, 2015.

[22] Roberts LJ, Huffam SE, Walton SF, Currie BJ. 2005 Crusted scabies: clinical and immunological findings in seventy-eight patients and a review of the literature. Journal of Infection, 50(5), 375–381. 15907543 10.1016/j.jinf.2004.08.033

[23] Savin JA. 2005 Scabies in Edinburgh from 1815 to 2000. Journal of the Royal Society of Medicine, 98(3), 124–129. 15738560 10.1258/jrsm.98.3.124 PMC1079418

[24] Sokolova TV, Radchenko MI, Lange AB. 1989 The seasonability of scabies morbidity and the fertility of the itch mite *Sarcoptes scabei* de Geer as an index of the activity of a population of the caucative agent. Vestnik Dermatologii i Venereologii, 11, 12–15. 2515675

[25] Thieberge G. 1922 Sur les variations de fréquence de la gale. Bulletin de l’Académie Nationale de Médecine, 88, 52.

[26] Tüzün Y, Kotoğyan A, Cenesizoğlu E, Baransü O, Ozarmağan G, Ural A, Cilara A, Gürler A, Tat AL. 1980 The epidemiology of scabies in Turkey. International Journal of Dermatology, 19(1), 41–44. 7358440 10.1111/j.1365-4362.1980.tb01993.x

[27] US Department of Health and Human Services, Public Health Service, Health Care Financing Administration. 1989 The International Classification of Diseases: 9th Revision, Clinical Modification: ICD-9-CM.

[28] Wang CH, Lee SC, Huang SS, Kao YC, See LC, Yang SH. 2012 Risk factors for scabies in Taiwan. Journal of Microbiology, Immunology and Infection, 45(4), 276–280. 10.1016/j.jmii.2011.12.003 22444547

[29] Zafar AB, Beidas SO, Sylvester LK. 2002 Control of transmission of Norwegian scabies. Infection Control & Hospital Epidemiology, 23(5), 278–279. 12026155 10.1086/502050

